# Antiviral effects of heme oxygenase-1 against canine coronavirus and canine influenza virus in vitro

**DOI:** 10.71150/jm.2501029

**Published:** 2025-05-27

**Authors:** Jae-Hyeong Kim, Dong-Hwi Kim, Kyu-Beom Lim, Joong-Bok Lee, Seung-Yong Park, Chang-Seon Song, Sang-Won Lee, Dong-Hun Lee, Do-Geun Kim, Hun-Young Yoon, In-Soo Choi

**Affiliations:** 1 Department of Infectious Diseases, College of Veterinary Medicine, Konkuk University, Seoul 05029, Republic of Korea; 2 Konkuk University Zoonotic Diseases Research Center, Konkuk University, Seoul 05029, Republic of Korea; 3 Korea Brain Research Institute, Daegu 41062, Republic of Korea; 4 Daegu Gyeongbuk Institute of Science and Technology, Daegu 42988, Republic of Korea; 5 KU Center for Animal Blood Medical Science, Konkuk University, Seoul 05029, Republic of Korea; 6 Department of Veterinary Surgery, College of Veterinary Medicine, Konkuk University, Seoul 05029, Republic of Korea

**Keywords:** antiviral, canine coronavirus, canine influenza virus, heme oxygenase-1

## Abstract

Heme oxygenase-1 (HO-1) has antioxidant, anti-apoptotic, and anti-inflammatory properties. Emerging evidence shows that HO-1 also exhibits antiviral activity against severe acute respiratory syndrome coronavirus 2, human immunodeficiency virus, hepatitis B virus, and Ebola virus. Its antiviral effects are mediated not only by its enzymatic function but also through the modulation of interferon-related pathways, thereby inhibiting viral replication. In this study, we investigated the antiviral effects of HO-1 on canine coronavirus (CCoV) and canine influenza virus (CIV) H3N2 using cell-based assays. To determine whether HO-1 suppresses CCoV and CIV, cells were treated with hemin to induce HO-1 expression. Hemin treatment successfully induced HO-1 expression in A72 and Madin-Darby canine kidney cells, resulting in the suppression of CCoV and CIV replication. The canine *HO-1* gene was cloned into an expression vector and transfected into cells to achieve transient overexpression. Recombinant canine HO-1 protein was expressed in *Escherichia coli* and purified using an expression vector. HO-1 overexpression suppressed CCoV and CIV replication in cells. Following viral infection, treatment with purified HO-1 protein led to a reduction in viral protein levels. Therefore, both HO-1 expression and exogenous protein treatment effectively inhibited CCoV and CIV replication. Elevated HO-1 protein levels consistently reduced viral RNA and protein expression in vitro. These findings suggest that HO-1 could serve as a potential therapeutic agent for managing viral infections in dogs.

## Introduction

Heme oxygenase-1 (HO-1) is encoded by the *HMOX1* gene and converts heme into carbon monoxide, ferrous ion, and biliverdin (Clark et al., 2000). As a cytoprotective enzyme, HO-1 is upregulated in response to cellular stress and contributes to antioxidant, anti-inflammatory, and anti-apoptotic responses (Facchinetti, 2020; Gozzelino et al., 2010; Waza et al., 2018). Recently, HO-1 has been investigated for its antiviral properties (Espinoza et al., 2017). Several studies have demonstrated that chemical inducers of HO-1 can suppress the replication of hepatitis B and C viruses (Lehmann et al., 2010; Protzer et al., 2007; Zhu et al., 2010). Additionally, HO-1 has been shown to inhibit the replication of other viruses, including Ebola virus (Hill-Batorski et al., 2013), dengue virus (Tseng et al., 2016), Zika virus (Huang et al., 2017), human immunodeficiency virus (Liu et al., 2016), and human respiratory syncytial virus (Che et al., 2023).

Canine coronavirus (CCoV) was initially reported in Germany in 1971 and has since become prevalent worldwide (Dong et al., 2022). The primary clinical manifestation of CCoV infection is gastroenteritis, typically presenting as diarrhea. Although this infection usually causes self-limiting enteritis, it can be severe in young puppies. In some cases, CCoV infection can result in fatal systemic illness, characterized by lethargy, anorexia, vomiting, bloody diarrhea, and ataxia (Alfano et al., 2020; Decaro and Buonavoglia, 2011; Radford et al., 2021).

Another significant canine pathogen, the H3N2 strain of canine influenza virus (CIV), was initially detected in Korea in 2007 (Dubovi, 2010). CIV H3N2 causes acute respiratory distress and severe morbidity. It has spread globally, with ongoing cases reported in several regions such as the United States and other Asian countries. Although CIV H3N2 typically causes mild respiratory illness, more severe diseases can occasionally occur (Chen et al., 2024; Janeczko, 2021; Voorhees et al., 2017).

Both CCoV and H3N2 are highly contagious viral pathogens in dogs. Despite the availability of commercial vaccines, infections continue to occur, often requiring hospitalization of affected dogs for treatment. The development of antiviral agents with broad-spectrum activity against multiple viruses might be useful in the management of infectious diseases in dogs. Our previous studies showed that hemin—a hemoglobin-degrading molecule—demonstrated in vitro antiviral activity against severe acute respiratory syndrome coronavirus 2 and hepatitis A virus (Kim et al., 2021a, 2021b). The antiviral activity of HO-1 in several viruses has been investigated, but studies examining its effects against canine viruses remain limited. Building on the findings of previous studies, this study aimed to evaluate the potential of HO-1 as a therapeutic candidate for the treatment of viral diseases in dogs.

In this study, a gastrointestinal disease-inducing CCoV and a respiratory disease-inducing CIV H3N2 were used to determine the antiviral activity of HO-1. HO-1 expression induced by hemin and an expression vector as well as treatment with recombinant HO-1 protein effectively inhibited viral replication in vitro.

## Materials and Methods

### Cell lines, viruses, and antibodies

A72 (ATCC no. CRL-1542) cells were used to propagate CCoV (ATCC no. VR-2068), whereas MDCK (ATCC no. CCL-34) cells were used to propagate CIV H3N2 (A/canine/Korea/LBM412/2008(H3N2)). Cell cultures were maintained in Dulbecco’s Modified Eagle Medium (DMEM) containing 4% heat-inactivated fetal bovine serum (FBS) (Gibco) and an antibiotic-antimycotic reagent (Gibco) under 5% carbon dioxide at 37°C. The following primary antibodies were used for immunoblotting and immunofluorescence assays (IFAs): anti-influenza A virus (IAV) (sc-130568, Santa Cruz Biotechnology), anti-CCoV (M938, BIOZOL Diagnostica), anti-glyceraldehyde-3-phosphate dehydrogenase (GAPDH) (ab8245, Abcam), anti-HO-1 (MA1-112, Thermo Fisher Scientific), and anti-His-tag (GTX110545, GeneTex).

### Cell cytotoxicity assay

Cell viability after treatment with hemin or recombinant canine HO-1 protein was assessed using the CCK-8 assay (Cat. 96992, Sigma-Aldrich). A72 and MDCK cells were seeded into 96-well plates, treated with designated concentrations of hemin or recombinant canine HO-1 protein, and incubated for 2 days. After removing the supernatant, the CCK-8 reagent was added to a serum-free medium and incubated for 4 h. Absorbance was measured at 450 nm to determine cell viability.

### Reverse transcription-quantitative polymerase chain reaction

Viral RNA or intracellular mRNA was extracted using an XENOPURE Total RNA Purification Kit (Cat. 93667873-TR, CELLTO BIO). The following primer sequences were used: CCoV-F, 5′-TTG ATC GTT TTT ATA ACG GTT CTA CAA-3′; CCoV-R, 5′-AAT GGG CCA TAA TAG CCA CAT AAT-3′; CIV-F, 5′-CAT TGG GAT CTT GCA CTT GAT ATT GTG GAT-3′; CIV-R, 5′-GAC AAA ATG ACC ATC GTC AAC ATC CAC AGC-3′; GAPDH-F, 5′-GGT CAC CAG GGC TGC TTT-3′; GAPDH-R, 5′-ATT TGA TGT TGG CGG GAT-3′; HO-1-F, 5′-GCG TCG ACT TCT TCA CCT TC-3′; and HO-1-R, 5′-GGT CCT CAG TGT CCT TGC TC-3′. One Step TB Green Green^®^ (RR086A, TaKaRa) was used for reverse transcription quantitative polymerase chain reaction (RT-qPCR) on a LightCycler (Roche). Cycling conditions were optimized based on previously published protocols (Lee et al., 2023a). Relative mRNA expression levels were normalized to the GAPDH level and calculated using the 2^-ΔΔCT^ method (Rao et al., 2013). All experiments were conducted in triplicate to ensure reliability.

### Plasmid transfection

The canine *HO-1* gene was amplified using PCR and cloned into a pcDNA3.1 (−) vector (V79520, Invitrogen). The constructed vector (pcDNA3.1 (−)/canine HO-1) or empty vector control (pcDNA3.1 (−)) was transfected into cells using Lipofectamine 2000 (Cat. 11668027, Thermo Fisher Scientific). Cells seeded into 6-well plates were transfected and incubated for 4 h. After incubation, the medium was replaced with DMEM supplemented with FBS.

### Recombinant protein purification

The coding sequence of canine HO-1 was inserted into the pET21a (+) vector (V011023, NovoPro). The resulting recombinant plasmid (pET21a (+)/canine HO-1) was transformed into BL21(DE3) cells (Wang et al., 2023). IPTG was added at a final concentration of 1 mM when the optical density at 600 nm reached between 0.6 and 0.8. Protein expression was induced for 5 h; next, the cells were harvested by centrifugation. The 6×His-tagged recombinant canine HO-1 protein was purified with HisTALON Gravity Columns (Cat. 635655, TaKaRa). Recombinant protein production was performed based on the method described by Kim et al. (2023), with modifications.

### Immunoblot assays

Immunoblot assays were performed according to the method described by Lee et al. (2023b). Cells were lysed in 2× Laemmli sample buffer (S3401, Sigma-Aldrich). The lysates were subjected to sodium dodecyl sulfate-polyacrylamide gel electrophoresis and subsequently transferred onto nitrocellulose membranes. After blocking with 5% skim milk (phosphate-buffered saline, 0.05% Tween 20), the membranes were incubated overnight with primary antibodies. Horseradish peroxidase-conjugated secondary antibodies were then added and incubated for 1 h. Signal detection was carried out using the SuperSignal^TM^ West Pico PLUS Chemiluminescent Substrate (Cat. 34580, Thermo Fisher Scientific).

### Immunofluorescence assay

IFAs were performed to detect HO-1 protein expression. Cells treated with hemin or recombinant canine HO-1 protein were analyzed 24 h post-treatment. IFAs were also conducted to detect CCoV and CIV proteins at 2 days post-infection. Cells were fixed with 4% paraformaldehyde and permeabilized with 0.5% Triton X-100 to detect intracellular viral proteins. The permeabilized cells were blocked with 1% bovine serum albumin and incubated with primary antibodies for 1 h. Subsequently, Alexa Fluor 488-conjugated secondary antibodies were added and incubated for 1 h in the dark. Nuclear counterstaining was performed using 4', 6-diamidino-2-phenylindole to visualize the nuclei. Fluorescence imaging was performed at 20× magnification using the EVOS FL Imaging System (AMF4300, Thermo Fisher Scientific). The fluorescence intensity was quantified using ImageJ software.

### Virus titration

Viral progeny production was determined using an endpoint dilution assay. A72 and MDCK cells were seeded in 96-well plates, and diluted virus supernatants were added. After incubation for 3–5 days, virus titers were calculated using the 50% tissue culture infectious dose (TCID_50_) method.

### Statistical analysis

All experiments were conducted in triplicate, and the results were expressed as means and standard deviation. Statistical analysis was performed using PRISM 8.0.1 (GraphPad Software, USA). Student’s *t*-test was employed for comparisons between groups. A p value of < 0.05 was considered significant.

## Results

### Purification and cellular delivery of recombinant canine HO-1 protein

**Fig. 1.**
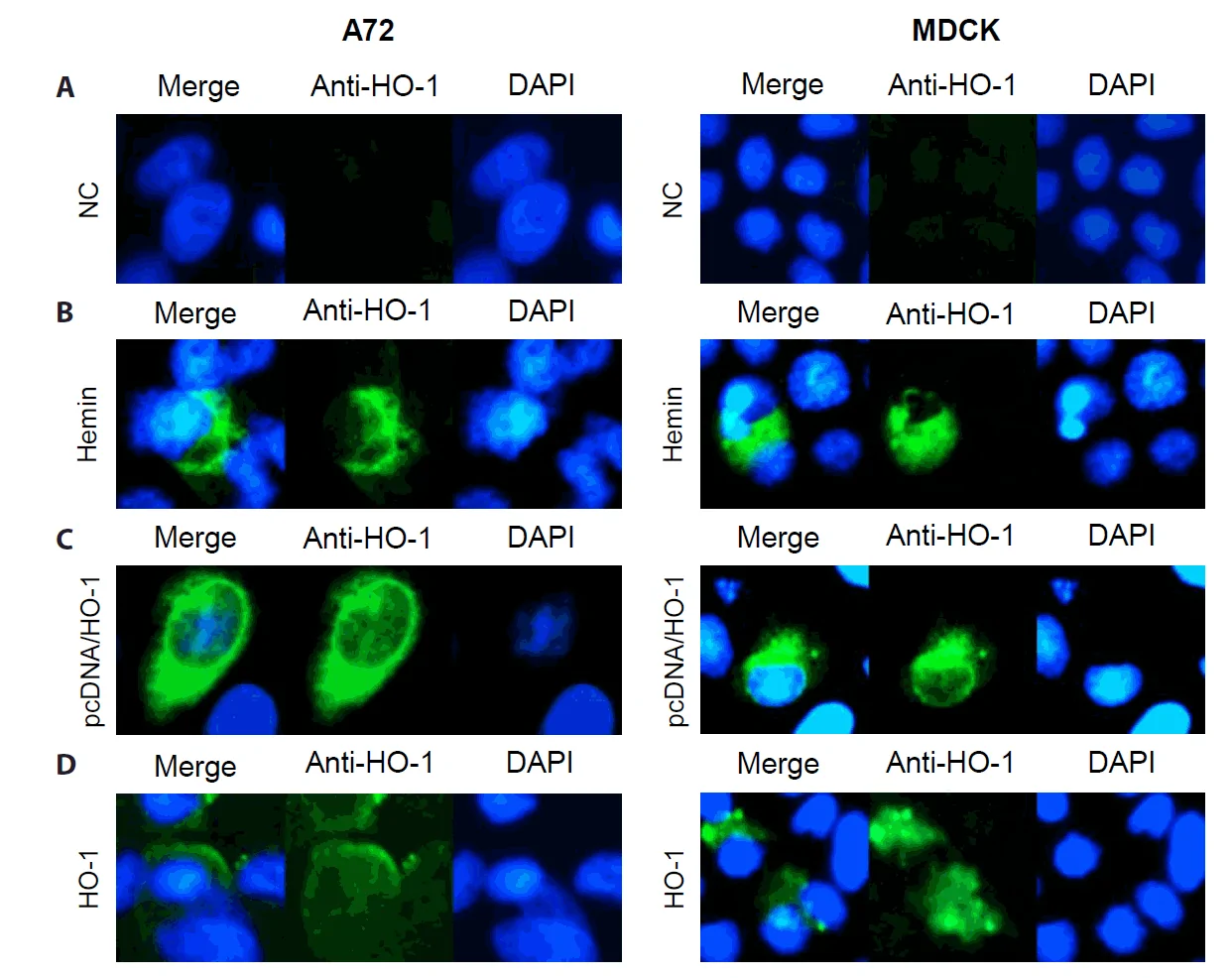
Detection of heme oxygenase-1 (HO-1) proteins in A72 and MDCK cells. HO-1 protein expression was confirmed through an immunofluorescence assay (IFA). IFA was conducted at 1 day after treatment. The expression of HO-1 protein in cells was visualized as green fluorescence, whereas nuclei were counterstained with DAPI. (A) Untreated cells were used as negative controls, with no significant HO-1 expression detected. (B) Cells treated with hemin exhibited HO-1 protein expression throughout the cytosol. (C) Cells were transfected with pcDNA 3.1(−) vector harboring the canine *HO-1* gene. (D) Cells treated with recombinant canine HO-1 protein showed a similar distribution pattern of HO-1 protein to that observed in the hemin-treated group. NC, negative control.

A72 and MDCK cells were treated with hemin. The dose-dependent increase in HO-1 mRNA expression level was confirmed through qPCR (Fig. S1), whereas HO-1 protein expression was confirmed through IFA. As shown in Fig. 1A and 1B, hemin treatment induced HO-1 protein expression in both A72 and MDCK cells. After 2 days of treatment, a significant increase in HO-1 mRNA levels was observed (Fig. S1). On the same day, cellular mRNA was extracted and amplified by PCR. The expression of HO-1 was detected in cells transfected with the vector encoding the canine *HO-1* gene (Fig. 1C). The recombinant HO-1 protein was confirmed by immunoblotting using anti-His-tag and anti-HO-1 antibodies (Fig. S2). The recombinant canine HO-1 protein was successfully delivered into the A72 and MDCK cells (Fig. 1D).

### Recombinant canine HO-1 protein showing no cytotoxicity in cells

**Fig. 2.**
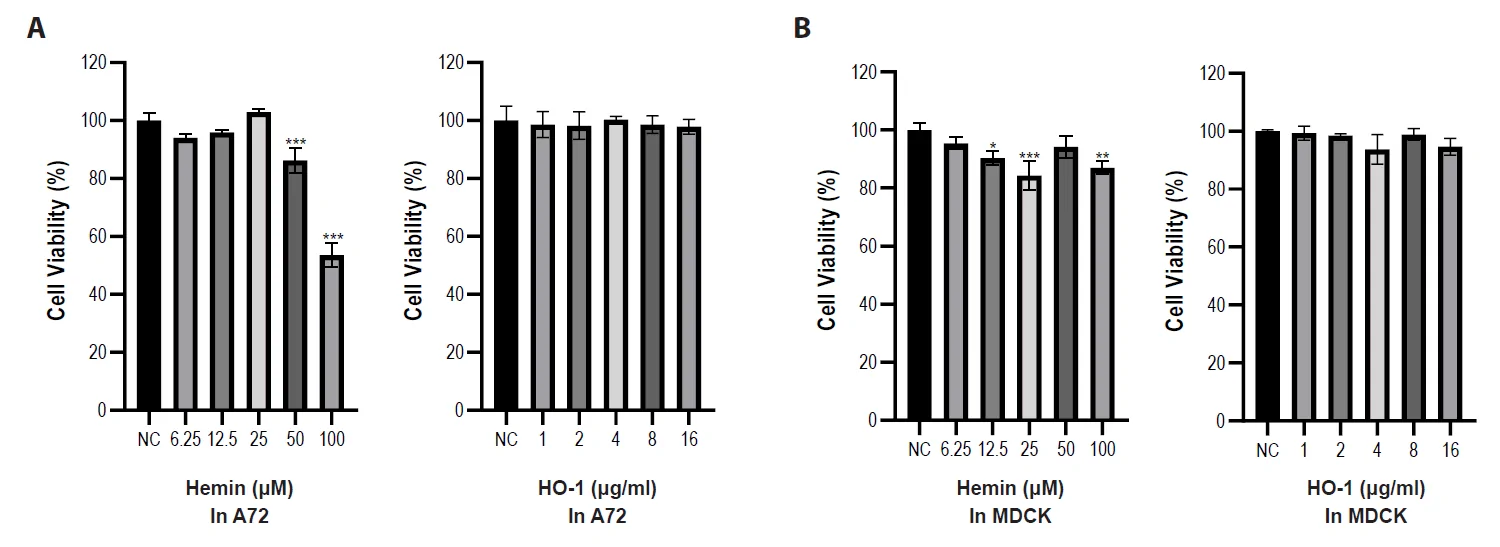
Effects of hemin and recombinant heme oxygenase-1 protein on cell viability in A72 and MDCK cells. Cell viability was assessed using a CCK-8 assay. (A) Cells were treated with various concentrations of hemin. Hemin showed higher cytotoxicity in A72 cells compared with MDCK cells. (B) Cells were treated with various concentrations of recombinant HO-1 protein. No significant cytotoxicity is shown in either cell at the tested concentrations. Untreated cells were used as negative controls. All experiments were performed in triplicate. Data are expressed as mean values, with error bars indicating the standard deviation. ^*^p < 0.05, ^**^p < 0.01, ^***^p < 0.001; NC, negative control.

Cytotoxicity assays were performed using hemin and purified recombinant canine HO-1 protein. Serial dilutions of hemin and HO-1 protein were added to the A72 and MDCK cells. Cell viability was assessed after 2 days and compared with that of untreated cells. As shown in Fig. 2, hemin showed cytotoxicity at concentrations ranging from 25 to 100 μM in both cell types. Therefore, 10 μM and 40 μM concentrations of hemin were selected for subsequent experiments. However, the recombinant HO-1 protein did not induce cytotoxicity at any of the tested concentrations (1–16 μg/ml) in both cell types. Therefore, HO-1 concentrations at 2, 4, and 8 μg/ml were chosen for further studies. We further examined the cytotoxicity of HO-1 using Annexin V staining. The results indicated no cytotoxicity in either cell line treated with 2, 4, and 8 μg/ml of HO-1 protein (Fig. S3).

**Fig. 3.**
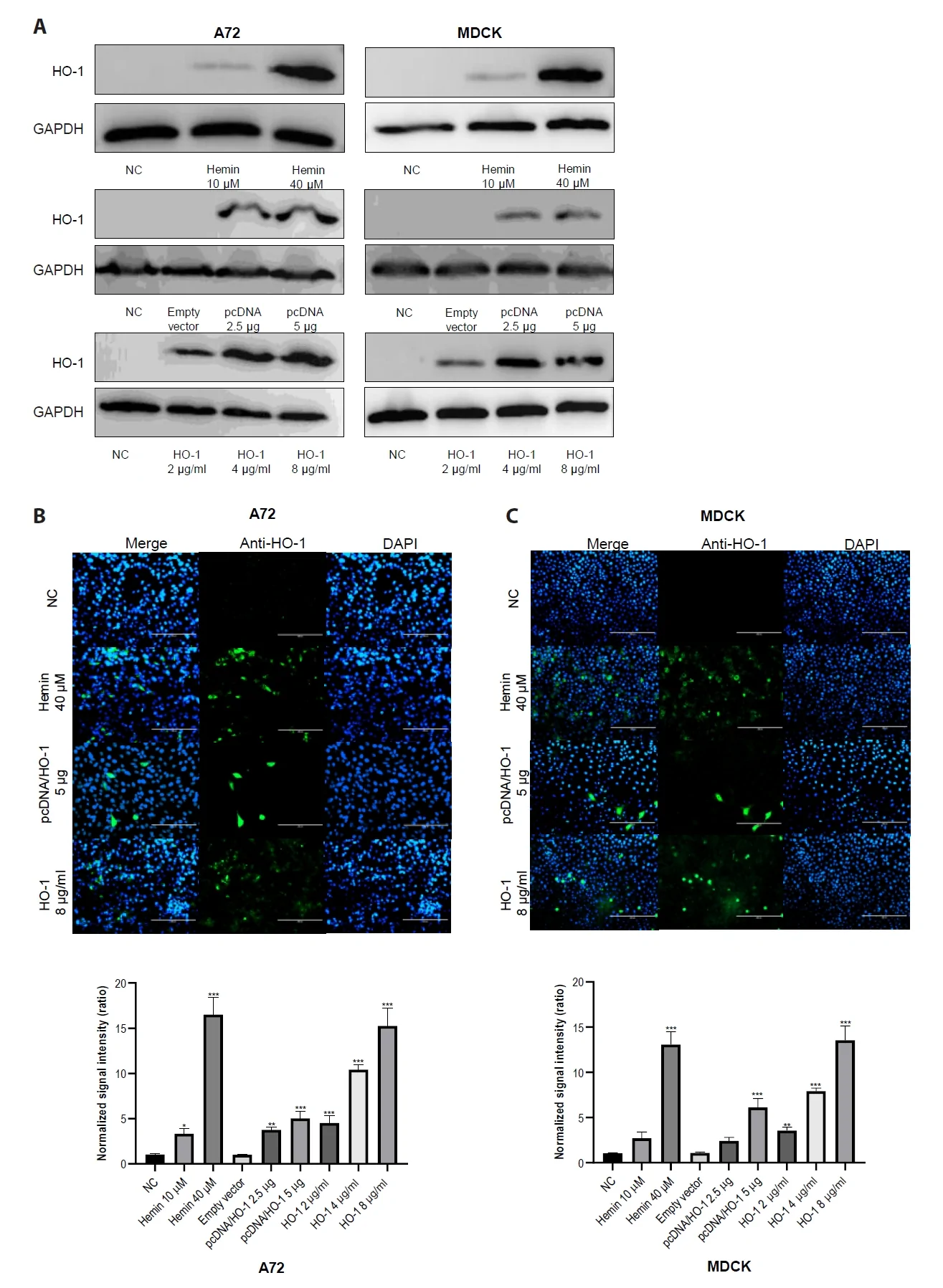
Analysis of heme oxygenase-1 protein expression using immunoblotting and immunofluorescence assay. (A) A72 and MDCK cells were treated with 10 or 40 μM of hemin. Cells were also transfected with either a pcDNA3.1 (−) harboring the canine *HO-1* gene or an empty vector. After 4 h of transfection, supernatants were removed and replaced with maintenance media containing fetal bovine serum. Cells were treated with recombinant HO-1 protein at concentrations of 2, 4, and 8 μg/ml. After 2 days of treatment, cells were harvested and analyzed. (B, C) HO-1 protein expression levels were analyzed using an IFA. Scale bar, 200 μm. (D) Quantitative data of fluorescence intensity were calculated using ImageJ software. Randomly selected four sites were analyzed to generate the graph. NC, negative control; empty vector, an empty pcDNA3.1 (−) vector.

After confirming the cytotoxicity test results, immunoblotting was performed to assess the changes in HO-1 protein levels in A72 and MDCK cells (Fig. 3). The cells were treated with hemin, pcDNA, or recombinant canine HO-1 protein, and the untreated cells served as negative controls. An increase in HO-1 protein expression was observed with increasing concentrations of hemin. As expected, elevated HO-1 protein expression was only detected in cells transfected with the pcDNA/HO-1 vector, whereas no increase was observed in cells transfected with the empty vector (Fig. 3A). Recombinant canine HO-1 protein was administered to both cell lines and maintained for 2 days after treatment. Successful intracellular delivery of HO-1 protein was confirmed by IFA (Fig. 3).

### Increase in HO-1 expression suppressing CCoV and CIV H3N2 viral RNA levels in vitro

**Fig. 4.**
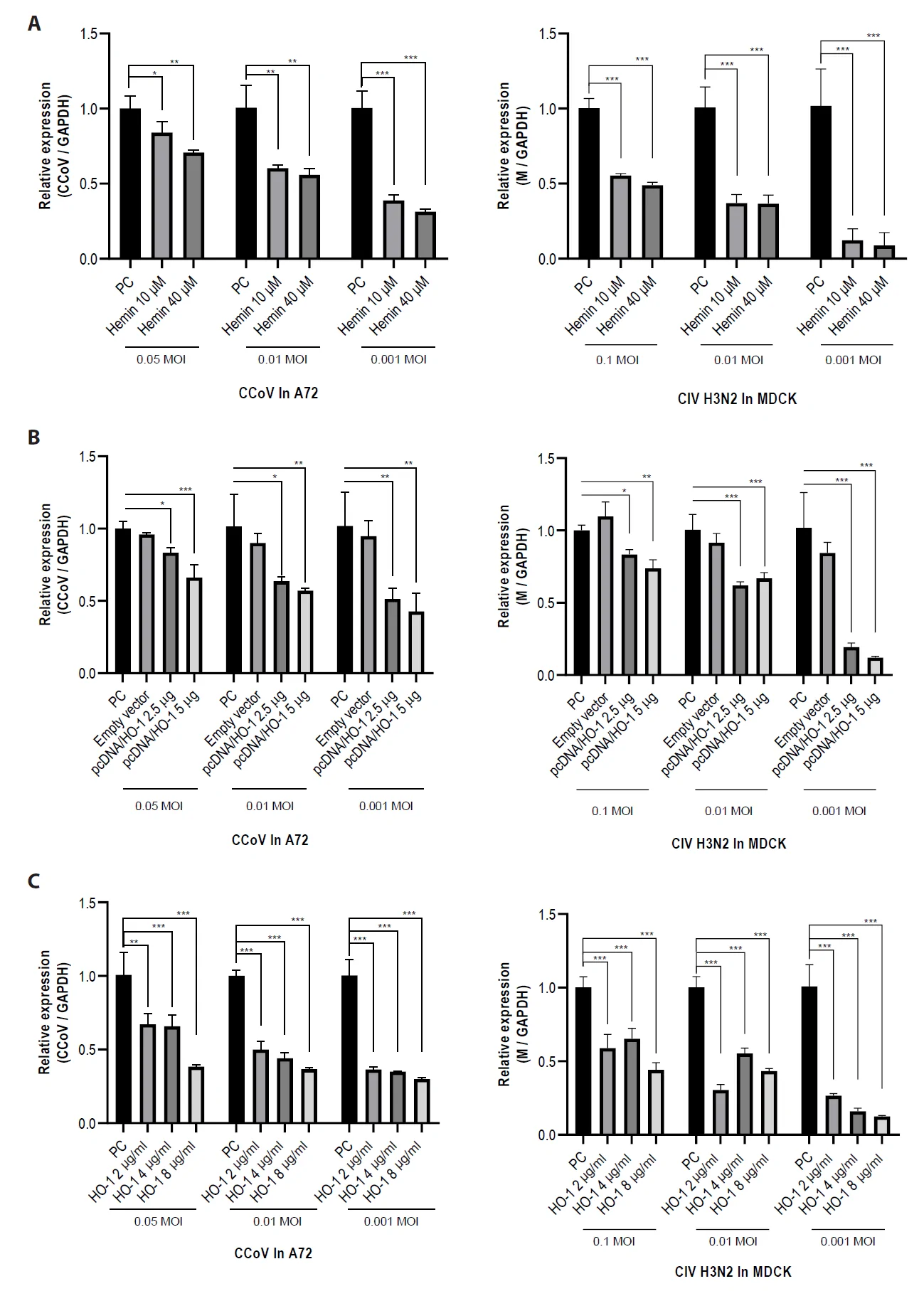
Inhibition of viral gene replication by increased expression of heme oxygenase-1. A72 and MDCK cells were infected with canine coronavirus (CCoV) and canine influenza virus (CIV) H3N2, respectively. (A) After viral infections, cells were treated with 10 or 40 μM of hemin. (B) Cells were transfected with either the pcDNA3.1 (−) harboring the canine *HO-1* gene (pcDNA/HO-1) or an empty vector prior to infection. (C) Cells were treated with recombinant canine HO-1 protein after infection. At 2 days post-infection, cells were harvested and lysed for mRNA analysis. The experiments were performed in triplicate. Data are expressed as mean values with error bars representing the standard deviation. ^*^p < 0.05, ^**^p < 0.01, ^***^p < 0.001; PC, positive control; empty vector, an empty pcDNA3.1 (−) vector.

RT-qPCR was conducted to evaluate changes in viral RNA and determine the antiviral activity of HO-1 against CCoV and CIV H3N2 (Fig. 4). A72 and MDCK cells were infected with CCoV and CIV H3N2, respectively. One hour after infection, the supernatant was replaced with a fresh medium containing various concentrations of hemin. At 2 days post-infection (dpi), cells were harvested, and intracellular viral RNA levels were analyzed. In the hemin-treated groups, the CCoV and CIV H3N2 RNA levels were reduced in a dose-dependent manner.

To determine whether the antiviral effect of hemin was attributable to the induction of HO-1 rather than its chemical properties, HO-1 was overexpressed using the pcDNA expression vector. A72 and MDCK cells were transfected with either pcDNA3.1 (−) harboring the canine *HO-1* gene (pcDNA3.1 (−)/canine HO-1) or an empty vector, using Lipofectamine 2000. After 4 h of transfection, the cells were infected with CCoV and CIV H3N2, respectively. At 2 dpi, the cells were harvested and analyzed for intracellular viral RNA. The results showed that the overexpression of HO-1 prior to viral infection attenuated viral replication. These results indicate that the antiviral activity of HO-1 could be manifested by its biological function rather than by the chemical properties of inducers such as hemin and cobalt protoporphyrin (CoPP).

To further explore the potential of HO-1 as an antiviral agent, recombinant canine HO-1 protein purified from *E. coli* was administered to cells following infection with CCoV and CIV H3N2. Cells were harvested at 2 dpi, and viral RNA levels were analyzed. A marked reduction in viral RNA levels was observed in both CCoV and CIV H3N2-infected cells treated with recombinant HO-1 protein.

### Suppression of CCoV and CIV H3N2 viral protein expression by HO-1 in vitro

**Fig. 5.**
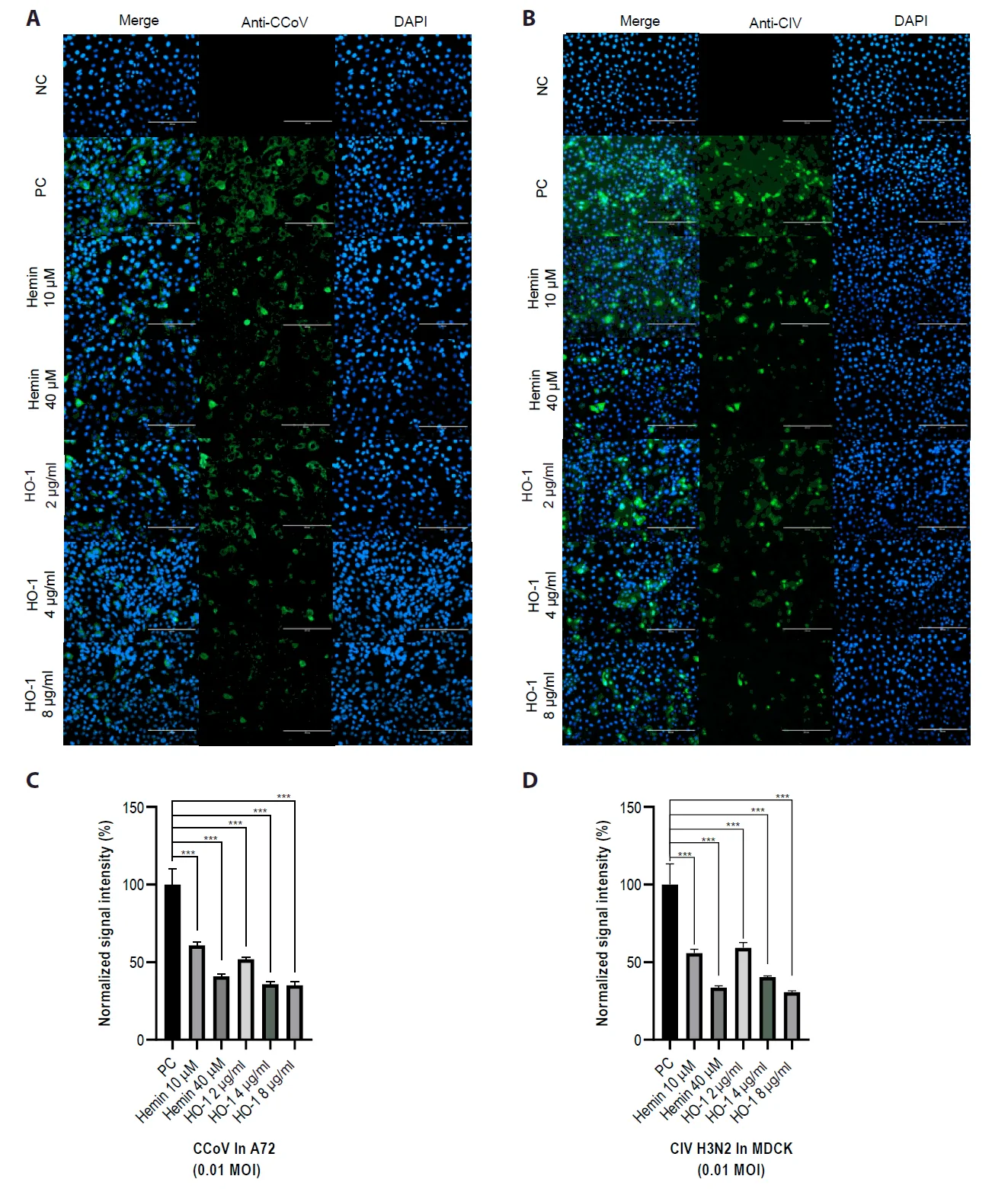
Reduction of viral protein expression following increased heme oxygenase-1 levels. Viral protein expression was assessed through an immunofluorescence assay (IFA). IFA was conducted at 2 days post-infection. Viral infection was visualized as green fluorescence. Nuclei were counterstained with DAPI. Scale bar, 200 μm. (A) Cells were infected with canine coronavirus (CCoV) or (B) canine influenza virus (CIV) H3N2. (C, D) Quantitative data of fluorescence intensity were calculated using ImageJ software. The experiments were performed in triplicate. Data are expressed as mean values with error bars showing the standard deviation. ^*^p < 0.05, ^**^p < 0.01, ^***^p < 0.001; NC, negative control; PC, positive control.

IFA was performed to determine the effect of HO-1 on viral protein expression in infected cells. At 2 dpi, IFA showed a reduction in CCoV or CIV H3N2 viral protein levels in association with increased HO-1 protein expression (Figs. 5 and S4). Green fluorescence, indicative of viral protein expression, was detected in cells infected with CCoV or CIV H3N2. Reduced fluorescence intensity was observed in cells treated with hemin or recombinant canine HO-1 protein.

Cells transfected with the pcDNA3.1 (−)/HO-1 vector were subsequently infected with CCoV or CIV H3N2. Two days after infection, viral protein expression was evaluated by IFA. The overexpression of HO-1 through transfection with the pcDNA3.1 (−)/canine HO-1 vector prior to viral infection resulted in the suppression of viral replication in both A72 and MDCK cells (Fig. S5).

### HO-1 suppressing CCoV and CIV H3N2 replication in vitro

**Fig. 6.**
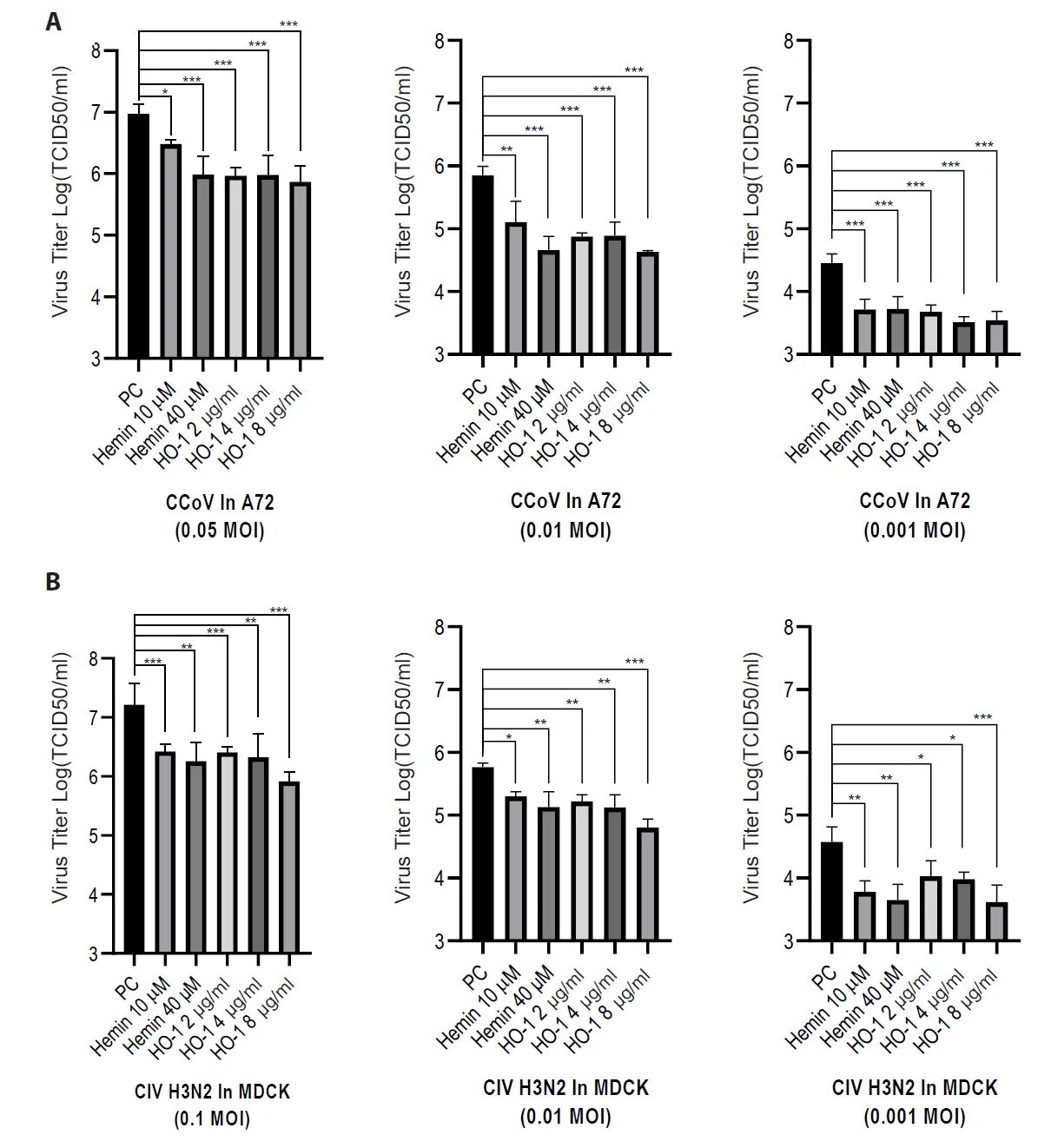
Effects of hemin and heme oxygenase-1 on viral replication. Viral titers were quantified by calculating the logarithm of the 50% tissue culture infectious dose [TCID_50_]). Cells were infected with diluted cell supernatants from the challenged groups at 2 days post-infection. (A) A72 cells were infected with canine coronavirus (CCoV), whereas (B) MDCK cells were infected with canine influenza virus (CIV) H3N2. The experiments were performed in triplicate. Data are expressed as mean values with error bars showing the standard deviation. ^*^p < 0.05, ^*^p < 0.05, ^**^p < 0.01, ^***^p < 0.001; PC, positive control.

Finally, the viral progeny in culture supernatants was quantified using an end-point dilution assay. A72 and MDCK cells were seeded in 96-well plates. Supernatants from infected groups were serially diluted in a culture medium. Each sample was added to cells, and the cells were incubated for 3–5 days. The Reed–Muench method was used to calculate the TCID_50_ (Figs. 6 and S6). The hemin- and recombinant canine HO-1 protein-treated groups showed reduced viral titers.

In summary, increased intracellular HO-1 protein levels suppressed CCoV and CIV H3N2 infections in vitro. HO-1, either induced by hemin or administered as a recombinant protein, reduced the viral RNA and protein levels. Additionally, increased HO-1 levels resulted in decreased virus titers in infected A72 and MDCK cells.

## Discussion

HO-1, as an inducible cytoprotective enzyme, is involved in heme metabolism and is activated in response to various stressors, including viral infections.

The present study demonstrated that HO-1 exhibits antiviral activity against CCoV and CIV H3N2 infections. Hemin was shown to suppress viral replication in vitro by inducing HO-1 expression. Several compounds, including CoPP-9 and andrographolide, induce HO-1 expression. Hemin was used in this study because of its established safety profile and approval by the United States Food and Drug Administration. It is commercially available as PANHEMATIN^®^ (Kim et al., 2021b).

To evaluate the HO-1’s specificity for antiviral activity, it was overexpressed using a pcDNA3.1(−) vector. In A72 and MDCK cells transfected with pcDNA3.1(−)/canine HO-1 vector, HO-1 expression was increased. The overexpression of HO-1 resulted in the suppression of viral replication in both cell lines. These findings suggest that the antiviral effect of hemin is mediated not only by its chemical properties but also by the biological activity of HO-1. Recombinant canine HO-1 protein purified from *E. coli* also inhibited viral replication in vitro.

The antiviral effects of HO-1 metabolites on viral infection have been investigated in several viruses, including porcine circovirus type 3 (Hou et al., 2023), porcine reproductive and respiratory syndrome virus (Wang et al., 2014; Zhang et al., 2017), bovine viral diarrhea virus (Zhang et al., 2015), pseudorabies virus (Zhang et al., 2020), and equid alpha herpesvirus 8V (Wang et al., 2024). Carbon monoxide, ferrous iron, and biliverdin, the downstream metabolites of HO-1, have been implicated in mediating its antiviral activity.

As demonstrated in several previous studies, HO-1 has a vital role in the regulation of innate immunity (Ma et al., 2019; Reichard et al., 2007; Ryter and Choi, 2016; Tzima et al., 2009). The activation of innate immune response before or during viral infection may enhance the host’s resistance to viral pathogens (Saha et al., 2024). Innate immunity—particularly the interferon (IFN) response—serves as an initial barrier encountered by viruses upon host cell entry. Mammalian cells have developed complex antiviral signaling pathways that lead to the induction of IFNs. These IFNs inhibit viral replication and trigger the adaptive immune response. Type I IFNs are essential for antiviral defense as they stimulate the expression of interferon-stimulated genes, which act synergistically to inhibit viral replication via multiple distinct pathways.

Many viruses, such as IAV, use viral proteins to evade or inhibit host innate immune responses. Strategies aimed at enhancing host cell immunity may help counteract viral infection (García-Sastre, 2011; Tecle et al., 2005). In addition to its direct antiviral effects mediated by its metabolites, HO-1 has also been implicated in the regulation of IFN production. The expression of HO-1 suppressed the replication of IAV through activation of the IRF3-mediated type I interferon pathway (Ma et al., 2019). These combined properties make HO-1 an attractive therapeutic target.

As demonstrated in numerous studies, viral replication can be inhibited by the overexpression of HO-1 through exogenous gene transfection (Kim et al., 2021a, 2021b; Wang et al., 2024; Zhang et al., 2020). In the present study, recombinant canine HO-1 protein was successfully expressed, purified, and used to directly treat cells. Consistent with the antiviral effects observed through HO-1 induction by hemin or gene overexpression, treatment with the recombinant protein also resulted in the suppression of viral replication in vitro.

Increased intracellular HO-1 protein levels were associated with reductions in viral RNA and protein levels in vitro. The antiviral activity against CCoV and CIV H3N2 may be attributed to the multifaceted cytoprotective effects of HO-1 and its metabolites, as reported in previous studies. These findings suggest that HO-1 holds promise as a potential therapeutic agent for the management of viral infections in dogs. Given its broad spectrum of biological activities, HO-1 may serve as a valuable treatment option in cases where specific antiviral agents or established therapies are lacking.

Although the current study demonstrates the antiviral effect against CCoV and CIV H3N2 occurs in vitro, further investigations are necessary to elucidate the specific mechanism underlying its action. Considering the diverse roles of HO-1 in innate immunity, additional studies are warranted to elucidate the antiviral mechanism of HO-1.

## Supplementary Information

Fig. S1. Induction of heme oxygenase-1 in cells. A72 and MDCK cells were treated with hemin at concentrations of 10 μM or 40 μM. After 2 days of treatment, cell lysates were analyzed to measure the HO-1 mRNA levels. HO-1 expression levels were compared with those of GAPDH and calculated using the 2^-ΔΔCT^ method. The experiments were performed in triplicate Data are expressed as mean values with error bars showing the standard deviation. ^*^p < 0.05, ^**^p < 0.01, ^***^p < 0.001. NC, negative control.

Fig. S2. Purification of the recombinant canine heme oxygenase-1 protein. The coding sequence of the canine *HO-1* gene was cloned into an expression vector. Recombinant proteins with 6-His tags were expressed and purified from *E. coli*. (A) Anti-His-tag antibody and (B) anti-HO-1 antibody were used as primary antibodies in the immunoblotting assays. Both antibodies detected recombinant canine HO-1 protein, with an approximate molecular weight of 32 kDa. Lanes: 1, cell lysates; 2, flow-through; 3, wash 1; 4, wash 2; 5, elution 1; 6, elution 2; M, marker.

Fig. S3. Annexin V staining. A72 and MDCK cells were treated with various concentrations of recombinant HO-1 protein and maintained for 2 days. After removing the supernatant, cells were stained with Annexin V (Annexin V-FITC Apoptosis Detection Kit, ab14085, Thermo Fisher Scientific) according to the manufacturer’s instructions. Counterstaining was performed using DAPI to visualize the nuclei. Cell death was evaluated using fluorescence microscopy (EVOS AMF4300, Thermo Fisher Scientific). (A) Cell death was induced in both A72 and MDCK cells. (B) A72 cells and (C) MDCK cells were treated with the recombinant HO-1 protein at various concentrations. The results showed no significant cytotoxicity in both cell lines. Scale bar, 200 μm. Quantitative fluorescence intensity data were obtained using ImageJ software. Randomly selected four spots were analyzed to generate the graphs. Data are presented as mean values with error bars showing the standard deviation. ^*^p < 0.05, ^**^p < 0.01, ^***^p < 0.001; NC, negative control.

Fig. S4. Viral protein detection through immunofluorescence assays at 2 days post-infection. Viral infection was visualized as green fluorescence. DAPI was used for nuclear counterstaining. Scale bar, 200 μm. (A) A72 cells were infected with canine coronavirus at 0.05 MOI, and (B) MDCK cells were infected with canine influenza virus H32N at 0.1 MOI. Cells were then treated with hemin or recombinant HO-1 protein at specific concentrations. (C, D) Quantitative fluorescence intensity data were obtained using ImageJ software. Four randomly selected spots were analyzed to generate the graphs. Data are expressed as mean values with error bars showing the standard deviation. ^*^p < 0.05, ^**^p < 0.01, ^***^p < 0.001; NC, negative control; PC, positive control.

Fig. S5. Immunofluorescence assay of the pcDNA3.1 (−)/canine HO-1-transfected group at 2 days post-infection. Viral infection was visualized as green fluorescence. DAPI was used for nuclear counter staining. Cells were transfected with the pcDNA3.1 (−)/canine HO-1 vector prior to viral infection. Scale bar, 200 μm. (A, B) A72 cells were infected with canine coronavirus at 0.05 or 0.01 MOI after transfection. (C, D) MDCK cells were infected with canine influenza virus H32N at 0.1 or 0.01 MOI after transfection. (E, F) Quantitative fluorescence intensity data were obtained using ImageJ software. Randomly selected four spots were analyzed to generate the graphs. Data are presented as mean values with error bars showing the standard deviation. ^*^p < 0.05, ^**^p < 0.01, ^***^p < 0.001; NC, negative control; PC, positive control; empty vector, an empty pcDNA3.1 (−) vector.

Fig. S6. Effects of pcDNA3.1 (−)/canine HO-1 transfection on viral replication. Viral titers were quantified by calculating the logarithm of the 50% tissue culture infectious dose [TCID_50_]). Cells were infected with diluted cell supernatants from the transfected groups at 2 days post-infection. (A) Cells were infected with canine coronavirus or (B) canine influenza virus H32N cells. The experiments were performed in triplicate. Data are expressed as mean values with error bars showing the standard deviation. ^*^p < 0.05, ^**^p < 0.01, ^***^p < 0.001; NC, negative control; PC, positive control; empty vector, an empty pcDNA3.1 (−) vector.
