# Supplementary material for: Antiviral effects of heme oxygenase-1 against canine coronavirus and canine influenza virus in vitro
Source: J Microbiol. 2025 May 27;63(5):e2501029. doi: 10.71150/jm.2501029 (PMC13577059; doi:10.71150/jm.2501029)

Fig. S1. Induction of heme oxygenase-1 in cells. A72 and MDCK cells were treated with hemin at concentrations of 10 μM or 40 μM. After 2 days of treatment, cell lysates were analyzed to measure the HO-1 mRNA levels. HO-1 expression levels were compared with those of GAPDH and calculated using the $2^{-\Delta\Delta CT}$ method. The experiments were performed in triplicate Data are expressed as mean values with error bars showing the standard deviation. *p < 0.05, **p < 0.01, ***p < 0.001. NC, negative control.

(a)

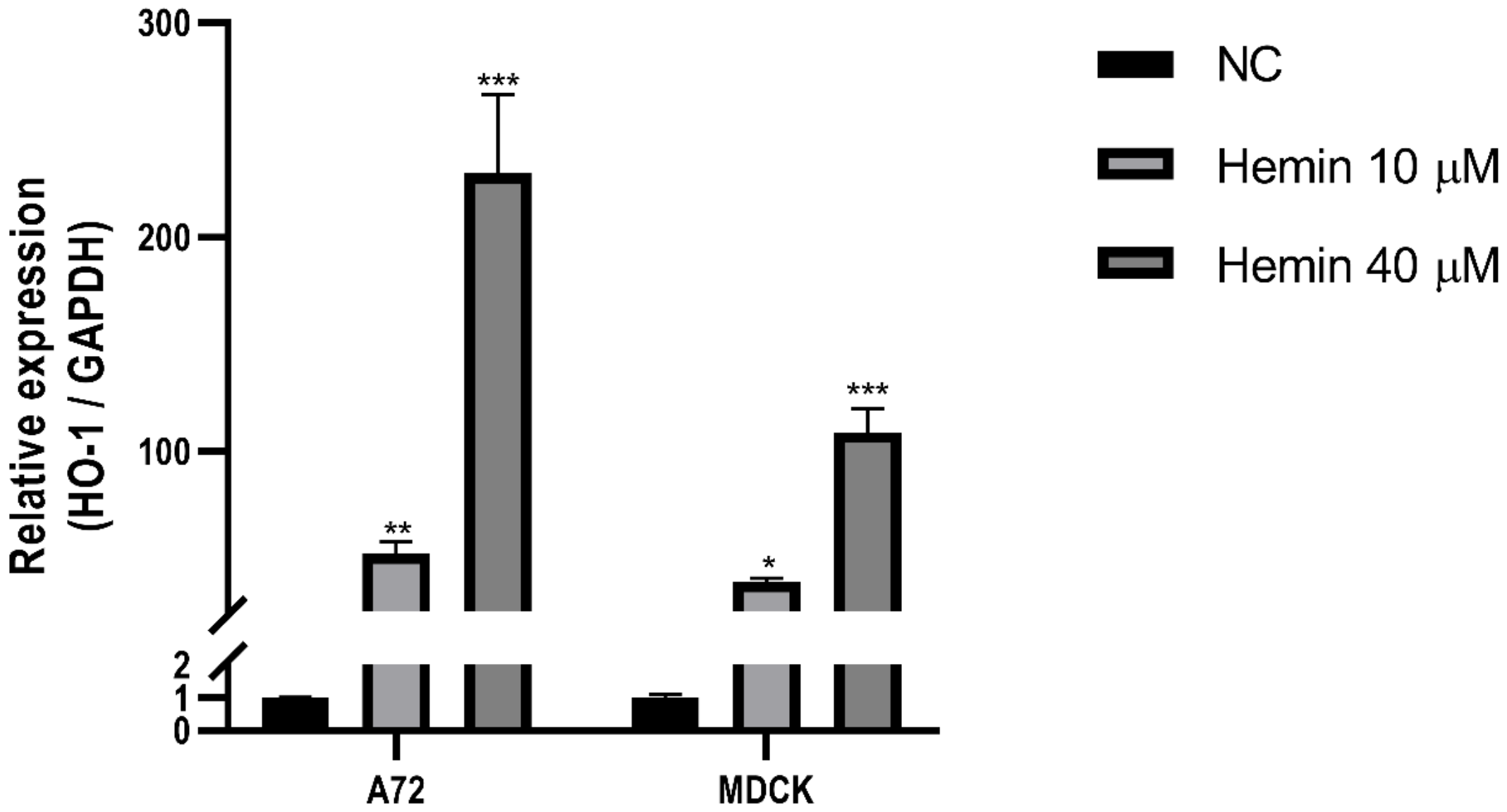

Supplement: Supplementary file 1 [file jm-2501029-Supplementary-Fig-S1.pdf]
