# Supplementary material for: Antiviral effects of heme oxygenase-1 against canine coronavirus and canine influenza virus in vitro
Source: J Microbiol. 2025 May 27;63(5):e2501029. doi: 10.71150/jm.2501029 (PMC13577059; doi:10.71150/jm.2501029)

Fig. S2. Purification of the recombinant canine heme oxygenase-1 protein. The coding sequence of the canine HO-1 gene was cloned into an expression vector. Recombinant proteins with 6-His tags were expressed and purified from *E. coli*. (A) Anti-His-tag antibody and (B) anti-HO-1 antibody were used as primary antibodies in the immunoblotting assays. Both antibodies detected recombinant canine HO-1 protein, with an approximate molecular weight of 32 kDa. Lanes: 1, cell lysates; 2, flow-through; 3, wash 1; 4, wash 2; 5, elution 1; 6, elution 2; M, marker.

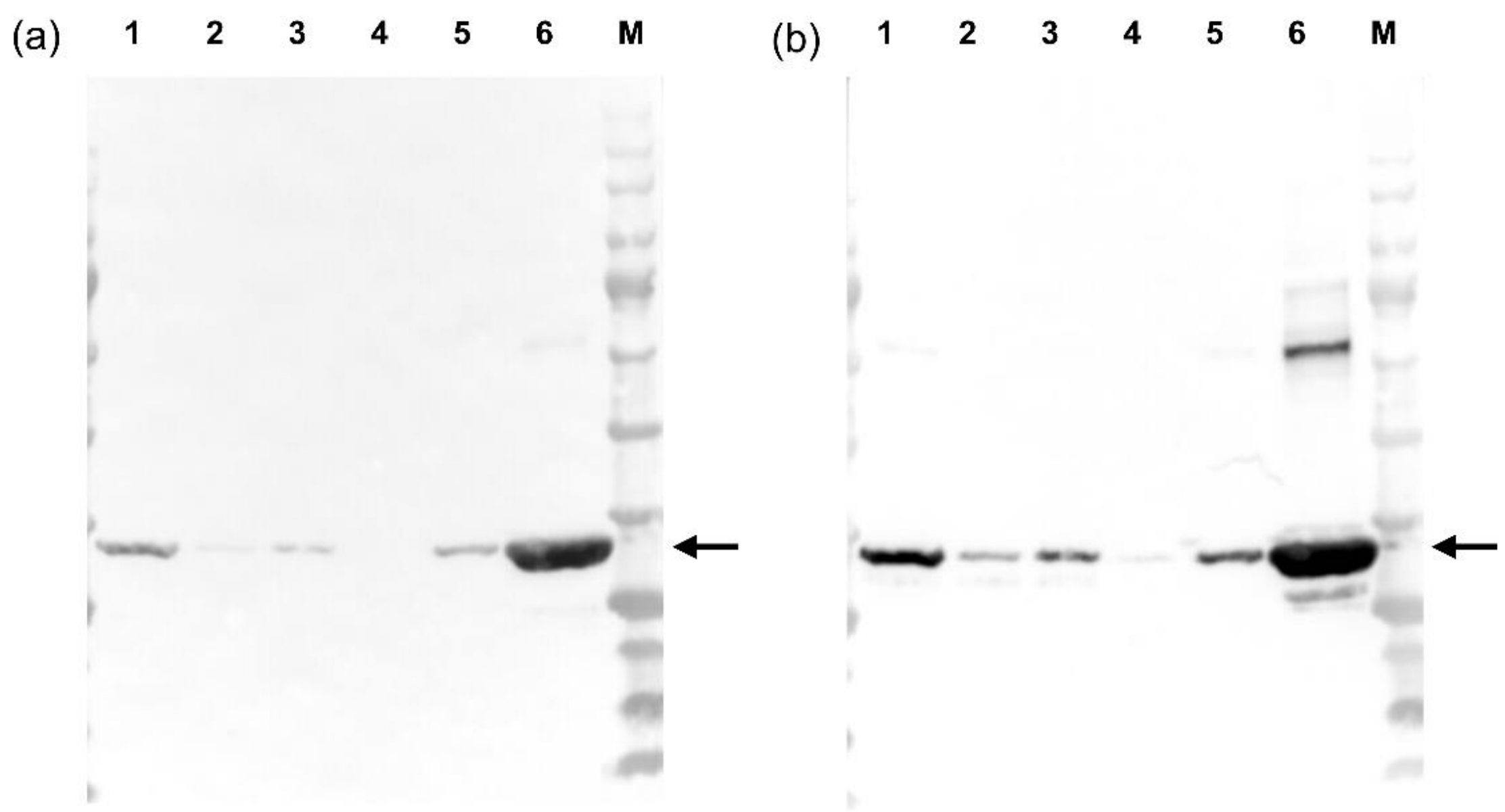

Supplement: Supplementary file 2 [file jm-2501029-Supplementary-Fig-S2.pdf]
