# Supplementary material for: Antiviral effects of heme oxygenase-1 against canine coronavirus and canine influenza virus in vitro
Source: J Microbiol. 2025 May 27;63(5):e2501029. doi: 10.71150/jm.2501029 (PMC13577059; doi:10.71150/jm.2501029)

Fig. S3. Annexin V staining. A72 and MDCK cells were treated with various concentrations of recombinant HO-1 protein and maintained for 2 days. After removing the supernatant, cells were stained with Annexin V (Annexin V-FITC Apoptosis Detection Kit, ab14085, Thermo Fisher Scientific) according to the manufacturer's instructions. Counterstaining was performed using DAPI to visualize the nuclei. Cell death was evaluated using fluorescence microscopy (EVOS AMF4300, Thermo Fisher Scientific). (A) Cell death was induced in both A72 and MDCK cells. (B) A72 cells and (C) MDCK cells were treated with the recombinant HO-1 protein at various concentrations. The results showed no significant cytotoxicity in both cell lines. Scale bar, 200 µm. Quantitative fluorescence intensity data were obtained using ImageJ software. Randomly selected four spots were analyzed to generate the graphs. Data are presented as mean values with error bars showing the standard deviation. $*p < 0.05$, $**p < 0.01$, $***p < 0.001$; NC, negative control.

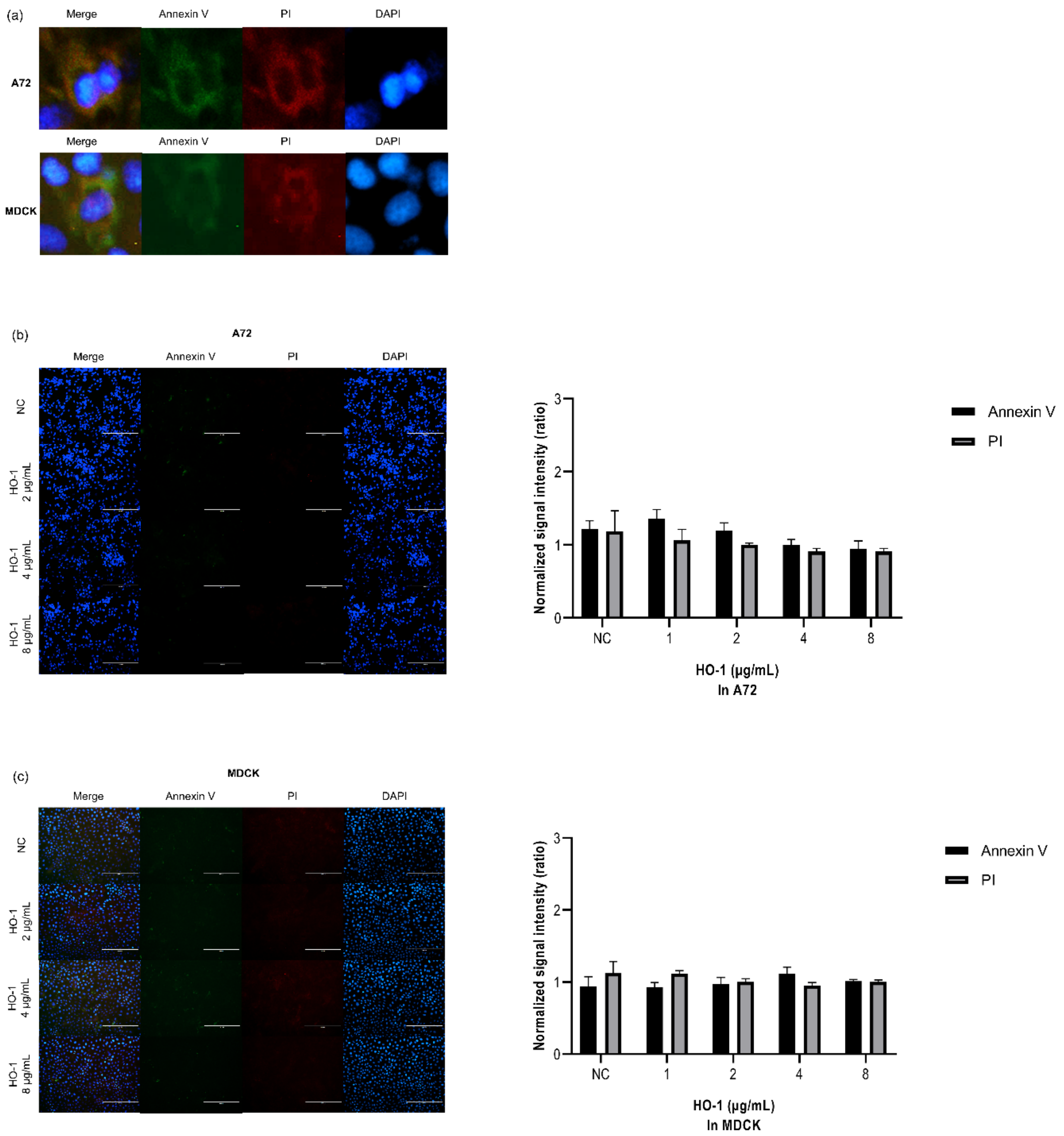

Supplement: Supplementary file 3 [file jm-2501029-Supplementary-Fig-S3.pdf]
