# Supplementary material for: Antiviral effects of heme oxygenase-1 against canine coronavirus and canine influenza virus in vitro
Source: J Microbiol. 2025 May 27;63(5):e2501029. doi: 10.71150/jm.2501029 (PMC13577059; doi:10.71150/jm.2501029)
Supplement: Supplementary file 4 [file jm-2501029-Supplementary-Fig-S4.pdf]

Fig. S4. Viral protein detection through immunofluorescence assays at 2 days post-infection. Viral infection was visualized as green fluorescence. DAPI was used for nuclear counterstaining. Scale bar, 200 µm. (A) A72 cells were infected with canine coronavirus at 0.05 MOI, and (B) MDCK cells were infected with canine influenza virus H32N at 0.1 MOI. Cells were then treated with hemin or recombinant HO-1 protein at specific concentrations. (C, D) Quantitative fluorescence intensity data were obtained using ImageJ software. Four randomly selected spots were analyzed to generate the graphs. Data are expressed as mean values with error bars showing the standard deviation. *p < 0.05, **p < 0.01, ***p < 0.001;

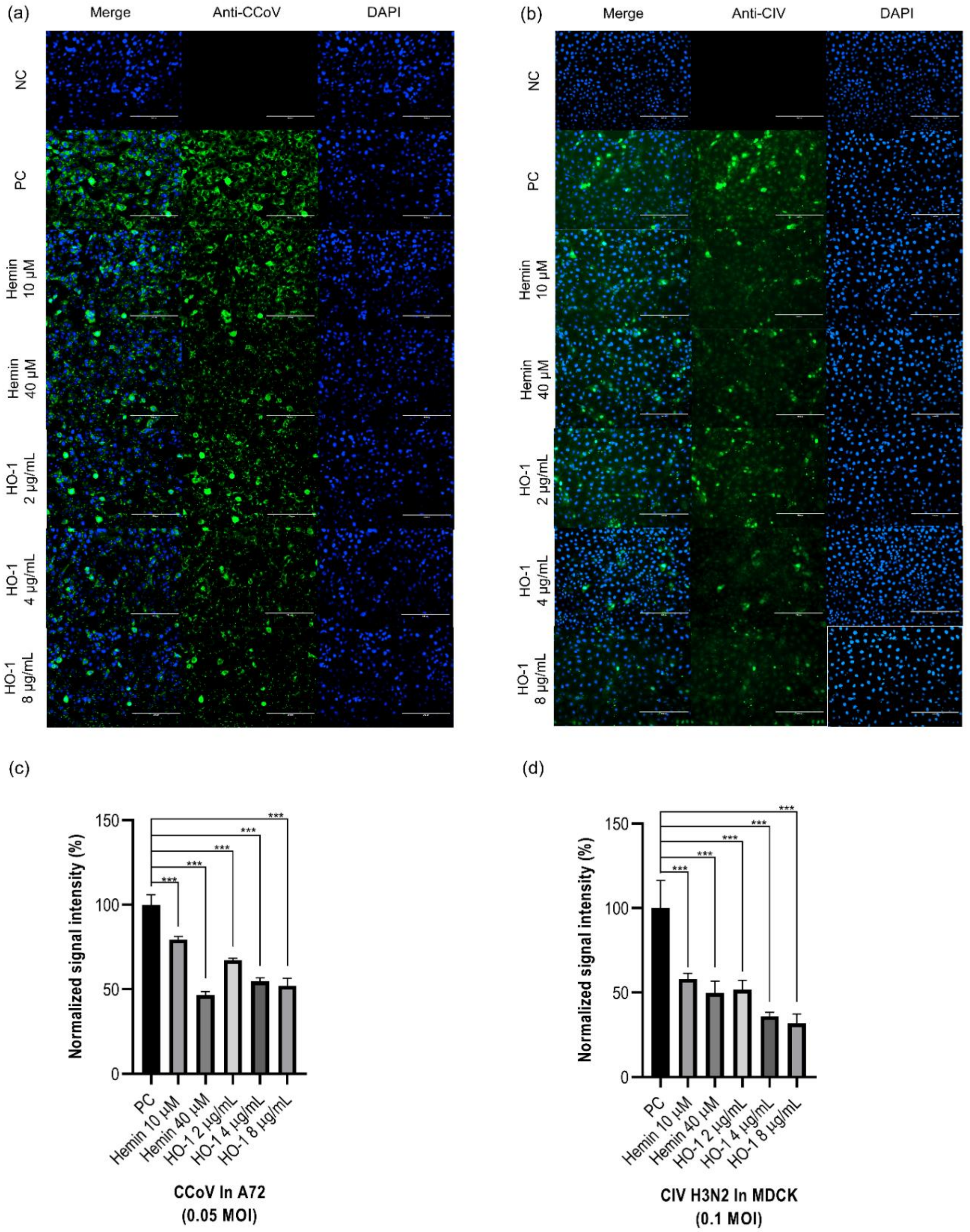

NC, negative control; PC, positive control.
