# Supplementary material for: Antiviral effects of heme oxygenase-1 against canine coronavirus and canine influenza virus in vitro
Source: J Microbiol. 2025 May 27;63(5):e2501029. doi: 10.71150/jm.2501029 (PMC13577059; doi:10.71150/jm.2501029)

Fig. S5. Immunofluorescence assay of the pcDNA3.1 (−)/canine HO-1-transfected group at 2 days post-infection. Viral infection was visualized as green fluorescence. DAPI was used for nuclear counter staining. Cells were transfected with the pcDNA3.1 (−)/canine HO-1 vector prior to viral infection. Scale bar, 200 µm. (A, B) A72 cells were infected with canine coronavirus at 0.05 or 0.01 MOI after transfection. (C, D) MDCK cells were infected with canine influenza virus H32N at 0.1 or 0.01 MOI after transfection. (E, F) Quantitative fluorescence intensity data were obtained using ImageJ software. Randomly selected four spots were analyzed to generate the graphs. Data are presented as mean values with error bars showing the standard deviation. *$p < 0.05$, **$p < 0.01$, ***$p < 0.001$; NC, negative control; PC, positive control; empty vector, an empty pcDNA3.1 (−) vector.

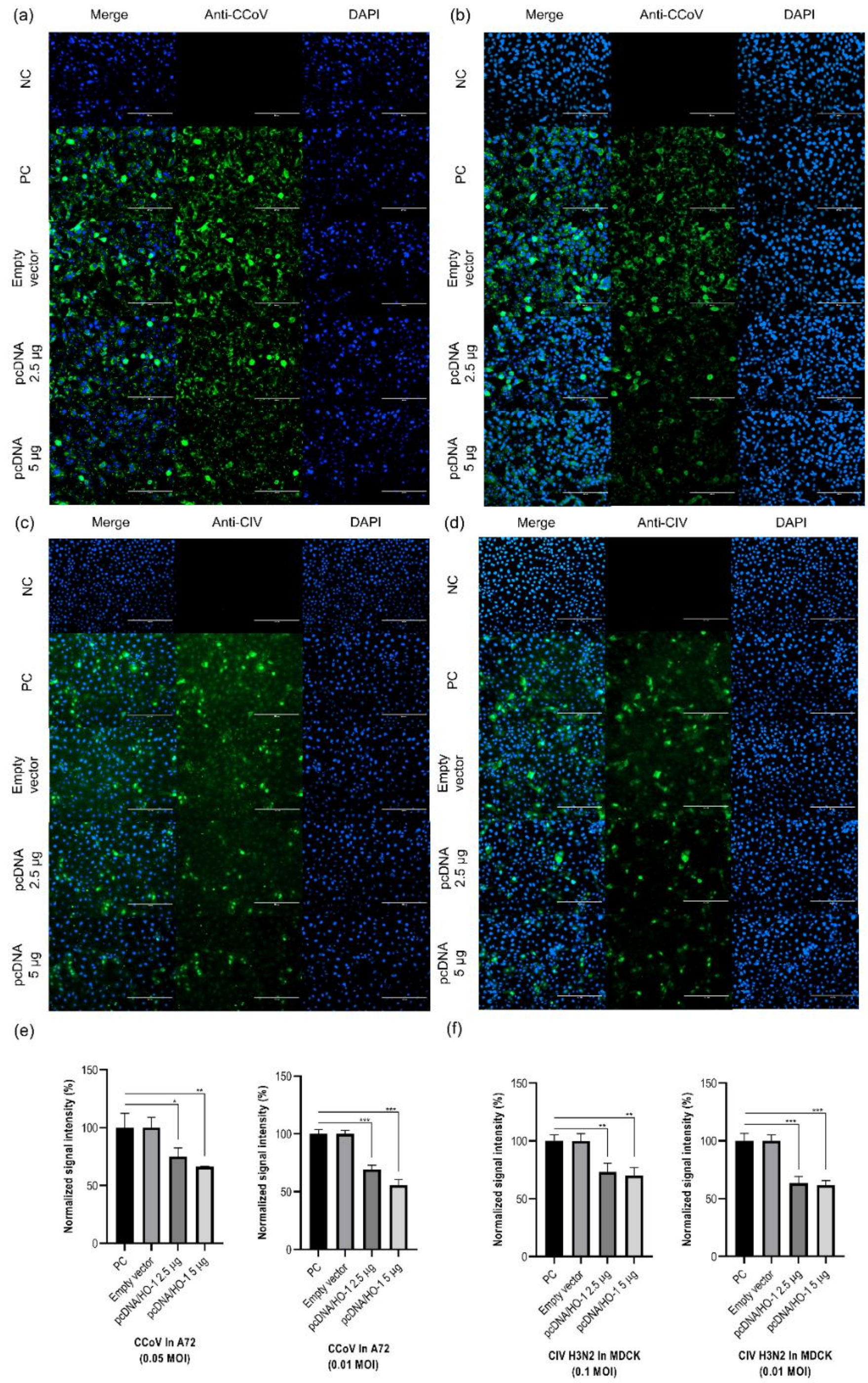

Supplement: Supplementary file 5 [file jm-2501029-Supplementary-Fig-S5.pdf]
