# Supplementary material for: Antiviral effects of heme oxygenase-1 against canine coronavirus and canine influenza virus in vitro
Source: J Microbiol. 2025 May 27;63(5):e2501029. doi: 10.71150/jm.2501029 (PMC13577059; doi:10.71150/jm.2501029)

Fig. S6. Effects of pcDNA3.1 (−)/canine HO-1 transfection on viral replication. Viral titers were quantified by calculating the logarithm of the 50% tissue culture infectious dose [TCID50]). Cells were infected with diluted cell supernatants from the transfected groups at 2 days post-infection. (A) Cells were infected with canine coronavirus or (B) canine influenza virus H32N cells. The experiments were performed in triplicate. Data are expressed as mean values with error bars showing the standard deviation. *p < 0.05, **p < 0.01, ***p < 0.001; NC, negative control; PC, positive control; empty vector, an empty pcDNA3.1 (−) vector.

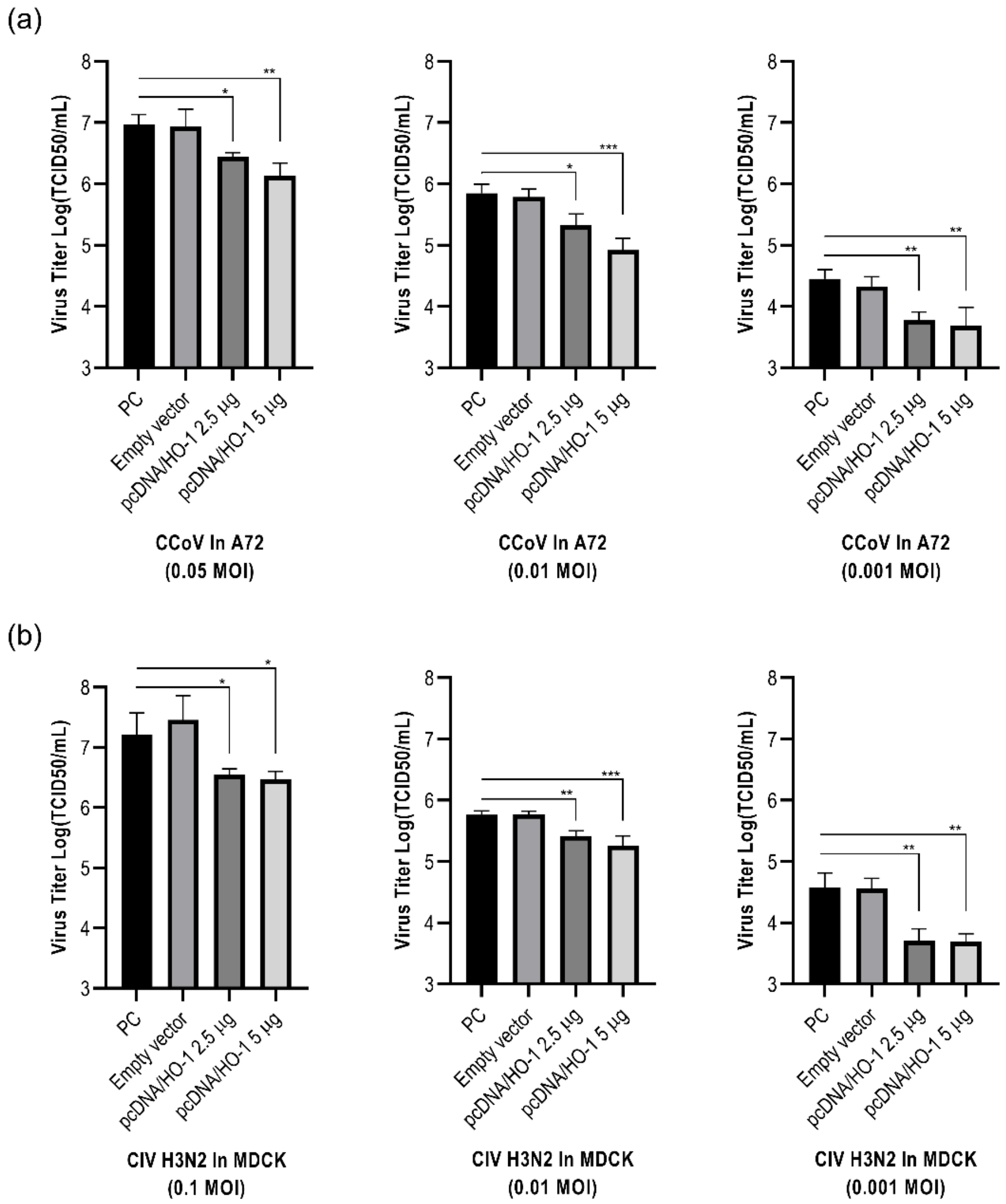

Supplement: Supplementary file 6 [file jm-2501029-Supplementary-Fig-S6.pdf]
